# TEMA and Dot Enumeration Profiles Predict Mental Addition Problem Solving Speed Longitudinally

**DOI:** 10.3389/fpsyg.2017.02263

**Published:** 2017-12-22

**Authors:** Clare S. Major, Jacob M. Paul, Robert A. Reeve

**Affiliations:** Melbourne School of Psychological Sciences, University of Melbourne, Melbourne, VIC, Australia

**Keywords:** dot enumeration profiles, longitudinal data analysis, school entry math ability, assessment and diagnosis, implications for intervention

## Abstract

Different math indices can be used to assess math potential at school entry. We evaluated whether standardized math achievement (TEMA-2 performance), core number abilities (dot enumeration, symbolic magnitude comparison), non-verbal intelligence (NVIQ) and visuo-spatial working memory (VSWM), in combination or separately, predicted mental addition problem solving speed over time. We assessed 267 children’s TEMA-2, magnitude comparison, dot enumeration, and VSWM abilities at school entry (5 years) and NVIQ at 8 years. Mental addition problem solving speed was assessed at 6, 8, and 10 years. Longitudinal path analysis supported a model in which dot enumeration performance ability profiles and previous mental addition speed predicted future mental addition speed on all occasions, supporting a componential account of math ability. Standardized math achievement and NVIQ predicted mental addition speed at specific time points, while VSWM and symbolic magnitude comparison did not contribute unique variance to the model. The implications of using standardized math achievement and dot enumeration ability to index math learning potential at school entry are discussed.

## Introduction

The question of which factors, or set of factors, best assess children’s math learning potential has long been of interests to educators. Standardized math tests (e.g., the Test of Early Math Ability – Ginsburg and Baroody, 1990, 2003) are commonly used for this purpose (Ginsburg and Baroody, 1990; Mazzocco and Myers, 2003; Mazzocco and Thompson, 2005; Halberda et al., 2008; Mazzocco et al., 2013; Ryoo et al., 2015). These tests provide a composite measure of different math abilities. Recent research also shows core number abilities, often indexed by the abilities to rapidly compare two Arabic numbers or non-symbolic quantities, or to name small sets of items, predict math abilities longitudinally (Reeve et al., 2012; Hornung et al., 2014). Core number abilities are thought to be a basis of children’s numerical competence. Furthermore, children’s math abilities have also been attributed to general cognitive factors (e.g., IQ, working memory) (Gray and Reeve, 2014, 2016). These sets of findings raise two questions: (1) do standardized math and core number abilities assess similar math abilities longitudinally; and (2) is the answer to the first question constrained by IQ or working memory? Answers to these questions may provide a framework for how best to assess math potential and design appropriate math interventions.

While standardized age-normed math tests may be helpful identifying children with math- learning disabilities, they have limitations (Mazzocco and Thompson, 2005; Mazzocco et al., 2013). Meaningful differences in performance among different math skills may be ignored and the contributions of core number skills underestimated (Geary et al., 2013). These limitations, in turn, may hinder the identification of math skills required for instruction and intervention (Geary et al., 2013). A composite math measure may show a child is falling behind age-related expectations, but not be the reason for the delay (Mazzocco et al., 2013). Understanding the factors that affect math delay is important since older children’s math abilities are dependent on earlier math abilities (Rittle-Johnson et al., 2001; Sarnecka and Carey, 2008).

Core number abilities are hypothesized to support early math development (Butterworth, 2010). A distinction is usually made between small precise number and approximate number abilities (Feigenson et al., 2004), both of which can be assessed in the young and are associated with numerical abilities. The ability to rapidly and precisely enumerate small sets, for example, predicts concurrent and future math achievement (Reeve et al., 2012; Sasanguie et al., 2013; Bartelet et al., 2014; Gray and Reeve, 2014). Dot enumerations tasks assess at least two distinct processes: a subitizing and a counting system, where small sets (*n* ≤ 4) are enumerated accurately and rapidly and larger sets (*n* ≥ 4) are enumerated more slowly and are more prone to counting errors (Schleifer and Landerl, 2011). Subitizing abilities are identifiable in infancy: Hyde and Spelke (2011), for example, identified brain signatures (event-related potentials) in pre-verbal infants, showing they respond to the precise cardinal value of small but not large sets.

In a 6-year longitudinal study, Reeve et al. (2012) identified distinct dot enumeration profiles in 5-year-olds and showed these signatures remained stable over the pre-high school years. Three signatures were identified that differed in subitizing range, subitizing slope and counting slope. Moreover, the profiles were associated with different math problems solving abilities over 6 years. A similar pattern of findings has been observed in preschoolers (Gray and Reeve, 2014, 2016).

Non-symbolic and symbolic magnitude comparison abilities are also claimed to reflect core number competence, and is typically assessed by judging which of two non-symbolic arrays (two sets of dots) or Arabic digits, is the larger. Some have found MC ability predicts future math achievement (De Smedt et al., 2009; Sasanguie et al., 2012). Desoete et al. (2012), for example, found preschoolers’ accuracy on a non- symbolic MC task predicted their arithmetic ability in first and second grade, independent of intellectual ability. However, Bartelet et al. (2014) found performance on a symbolic, but not on a non- symbolic, MC task predicted first grade arithmetic, controlling for intelligence and processing speed. Moreover, Vanbinst et al. (2014) showed performance on a symbolic MC task at the start of schooling predicted math ability, independent of general cognitive functioning and school-entry mathematical ability.

Working memory is associated with young children’s math ability (Bull et al., 2008; Holmes et al., 2008; Gray and Reeve, 2014; Scüzs et al., 2014). Visuo-spatial working memory (VSWM) appears important in the early stages of arithmetic learning (Barrouillet and Lépine, 2005; Imbo and Vandierendonck, 2007; De Smedt et al., 2009; Friso-van den Bos et al., 2013). A relationship between working memory and math is not always found, however (Passolunghi and Siegel, 2004; Meyer et al., 2010; Peng et al., 2016). In a recent meta-analysis Peng et al. (2016) note the relationship between WM and math performance appears dependent, in part, on the nature of the WM and math tasks used in research (see also Lan et al., 2011; Purpura and Ganley, 2014; Purpura et al., 2017; Schmitt et al., 2017).

Non-verbal intelligence (NVIQ) also appears to be related to math abilities and is a commonly used measure of math achievement (Scüzs et al., 2014). In a longitudinal study Geary et al. (2017) found NVIQ was a stable predictor of children’s math achievement over time (Van de Weijer-Bergsma et al., 2015; Lee and Bull, 2016; Tolar et al., 2016). One explanation for this association is NVIQ, in part, requires visuo-spatial organization abilities necessary for early math problem-solving ability (Rourke, 1988; Scüzs et al., 2014).

In the present study we investigated children’s mental addition problem solving speed longitudinally. The rationale for focusing on mental addition problem solving speed is it has been found to be closely associated with the strategy employed to solve problems. The problem solving strategies children employ to solve single addition problems skills, on average, change in their conceptual sophistication over time and are claimed to represent changes in math reasoning abilities (Butterworth, 2005; Reeve et al., 2012; Paul and Reeve, 2016). With age and/or experience, children change from using less sophisticated strategies (e.g., guess→ Count All →Count On →Min Count) to more sophisticated strategies (Decomposition→ Retrieval) (Donlan et al., 2003; Geary et al., 2004, 2007). The speed and accuracy with which children solve single digit addition problems is highly correlated with their strategy sophistication (Canobi et al., 1998, 2002; Paul and Reeve, 2016). For example, a Count All strategy, where each addend is individually enumerated, takes more time to execute and is more error prone than a Retrieval strategy where answers are retrieved from memory (i.e., the answer is known). Moreover, links have been found between single digit addition abilities and other math-related factors, working memory and core number abilities (Reeve et al., 2012; Paul and Reeve, 2016).

### The Current Study

In the present study we investigated whether the TEMA-2, magnitude comparison, dot enumeration, VSWM, assessed at school entry, and NVIQ abilities, assessed at 8-years, similarly or differently predict addition problem-solving RTs across time. We selected the TEMA because it assesses a range of math skills, has been used to identify math learning trajectories (Kable et al., 2015; Chu et al., 2016), and has been normed for use with young children, which is regarded as particularly useful (Feigenson et al., 2013). Given that the TEMA is frequently use as a standardized measure for determining children’s math trajectories (Purpura et al., 2015), an examination of its performance in predicting math outcomes longitudinally seems sensible. Moreover, comparing TEMA’s performance with other core math measures would be advantageous in supporting the development of math assessment schedules in which assessment tools are used in a ‘best-fit-for-purpose’ manner.

To address this issue, 267 5 to 6-year-olds’ abilities were assessed on four occasions over a 5-year period. The dot enumeration and magnitude comparison measures were selected because individuals’ performance is relative stable over time (Reeve et al., 2012). The addition measures were selected because of their importance in children’s school curriculum (Paul and Reeve, 2016; Australian Curriculum Assessment and Reporting Authority, 2017). Addition measures were tailored to reflect children’s expected age-related math competence, as articulated in the national curriculum guidelines. Single-digit mental addition problem solving was assessed in 6 to 7-year-olds, single and double-digit mental addition was assessed in 8 to 9-year-olds, and double-digit addition requiring decomposition was assessed in 10 to 11-year olds.

We used longitudinal path analysis to identify the factor, or combination of factors, that best predicted addition RTs across time. Of interest was the degree to which dot enumeration, magnitude comparison abilities and/or TEMA test scores, assessed at school entry, moderated by VSWM and NVIQ, independently or in association, predicted mental addition problem solving RTs 2, 4, and 6 years later. On the basis of previous research findings we expected TEMA scores, dot enumeration profile membership and magnitude comparison RTs to predict mental addition RTs across time. We also expected that NVIQ and VSWM would moderate the impact of the TEMA and two core number measures. Of particular interest was the degree to which the TEMA, dot enumeration profile membership and magnitude comparison RTs (1) contributed unique variance in predicting mental addition RTs, and (2) predicted mental addition RTs in the short and long term.

## Materials and Methods

### Participants

Two hundred and sixty-seven children (*M* = 72.49 months, *SD* = 4.50 months at the beginning of the study), comprising 156 boys (*M* = 72.98 months, *SD* = 4.39 months) and 111 girls (*M* = 71.79 months, *SD* = 4.57 months), attending schools in middle-class suburbs of a large Australian city, participated in the study. The data were collected as part of a larger study examining the development of math ability in preadolescent children over time (see Reeve et al., 2012). The data described herein were collected on four different occasions over a 5-year period (hereafter referred to as Time 1, Time 2, Time 3, and Time 4). Time 1 and Time 2 were separated by approximately 12 months; and Time 2 and Time 3, and Time 3 and Time 4, were separated by approximately 24 months respectively. All children spoke English fluently, had normal or corrected to normal vision and according to school personnel, had no known learning disabilities. The study was conducted with the agreement of, and in compliance with, the authors’ University’s Human Ethics Committee. Parents gave written consent allowing their child to participate in the project.

### Materials and Procedure

Children were tested toward the beginning of their school year. They completed the dot enumeration (DE), magnitude comparison (MC), VSWM, and TEMA-2 at Time 1. The TEMA-2 and the VSWM task were completed on the first day, and the DE and MC tasks, on consecutive days. Children completed a single-digit addition test at Time 2; a mixed addition test at Time 3; and a double-digit addition test at Time 4. They completed the non-verbal IQ test at Time 3. Test sessions lasted between 15 and 20 min. On occasions where more than one task was completed in a single test session, task presentation order was randomized. The TEMA was selected because of its high test–retest reliability (α = 0.94: Ginsburg and Baroody, 1990), as was the non-verbal IQ and VSWM measures (VSWM: Corsi Blocks test, α = 0.75: de Paula et al., 2016; and the Ravens Progressive Matrices non-verbal IQ test, α = 0.82: Cotton et al., 2005).

The dot enumeration, magnitude comparison tasks and addition tasks were presented on a 15″ laptop computer, running DMDX (Version 2) software that controlled stimuli presentation and allowed RTs to be recorded. Responses were recorded by an interviewer pressing a response key. This procedure was preferred over a voice activated recording system to avoid the impact of children counting aloud, which would terminate a voice activated recording system. The interviewer was unaware of what appeared on the screen. Test items were presented in a random order.

#### Dot Enumeration

The dot enumeration task comprised dot arrays comprising one to nine black dots (0.2 cm in diameter) presented on a white background. Dots were randomly positioned within a 15 cm × 11 cm grid and were no less than 2 cm apart (to reduce the appearance of clustering). Each dot numerosity was presented eight times (*n* = 72 trials overall). Children completed five practice trials in which they reported as quickly as possible the number of dots in each array (Tell me as fast as you can – but without making any mistakes – how many dots you see on the screen). Instructions were repeated to ensure children understood task requirements. Responses were scored as correct or incorrect, and RTs recorded.

Dot enumeration abilities were determined using Reeve et al.’s (2012) four-parameter model. The model is derived from a latent profile analysis of children’s RTs to one to five dots and an average of six to eight dots. We analyzed RTs (for correct responses) for one, two, three, four, and five dots and the average of six to eight dots to differentiate between the subitizing range and slope, the counting slope and the point of discontinuity between the subitizing and counting ranges. Specifically, by analyzing the nature of the change in children’s RT slope function in one to five dots, we were able to identify the point of inflection in the RT slope and hence the subitizing range, slope and intercept, as well as the counting slope. The latent profile analysis was based on these data, which yielded three distinct response profiles distinguishable in terms of differences in (1) subitizing range, (2) subitizing slope, and (3) counting slope (see Reeve et al., 2012 for details). For convenience, here we refer to these profiles as the “slow,” “medium,” and “fast” profile. (It should be noted that while children in the profile differed in overall response speed, they also differed in subitizing range and differences in subitizing and counting slopes RTs.) The analyses reported here are based on these profiles.

#### Magnitude Comparison

The magnitude comparison task comprised single-digit pairs. Children pressed the left shift key if the number on the left side of the screen was large or the right shift key if the number on the right side of the screen was larger. All combinations of numbers 1 to 9 were presented, giving a total of 72 trials. Children were presented with practice problems to familiarize them with the task. Responses were scored as correct or incorrect, and RTs recorded. However, because error rates were low, and there were no RT differences between correct and incorrect responses, analyses were based on overall mean RTs.

#### Visuo-spatial Working Memory (VSWM)

Visuo-spatial working memory was assessed using the Corsi Blocks task (Milner, 1971). It was administered and scored following Kessels et al.’s (2000) procedure. VSWM span was calculated as the average of the longest correct block tap sequences. Spearman correlation measured across both trials ρ = 0.48, *p* < 0.001, indicates high reliability.

#### Non-verbal IQ (NVIQ)

Non-verbal IQ was assessed using the Ravens Colored Progressive Matrices test. It was administered following manual instructions (Raven et al., 1984) and scored using standardized norms (Raven et al., 1998). Good inter-item consistency and split-half reliability have shown in comparable samples of Australian school children (Cotton et al., 2005). The reliability estimate for our sample was good (α = 0.82).

#### Test of Early Mathematical Abilities Version 2 (TEMA-2)

General math ability was assessed by the TEMA (Ginsburg and Baroody, 1990). While the TEMA is normed on United States children, it is widely used in other countries (Ginsburg and Baroody, 1990). It has a high reported internal consistency (coefficient reliability, α = 0.94) for the age of our sample (Mazzocco and Thompson, 2005). The reliability for our current sample was calculated as α = 0.91. The TEMA-2 was selected in preference to the TEMA-3, as increases in the number of test items in the TEMA-3 significantly increased administration time (up to 20 min extra) and was deemed unwarranted [The TEMA-2 assesses seven abilities: counting using one-to-one correspondence; verbal counting—counting with number words; numerical comparison—comparing the magnitudes of sets (e.g., Which side has more?); set construction—constructing different sets of items (e.g., Can you show me six?); numeral literacy—recognizing and writing whole numbers (e.g., What number is this?); number facts—computing fluently with basic numbers (e.g., How many is one less than two?); calculation—addition, subtraction and multiplication (e.g., How many points does he have altogether?) It should be noted that the comparing magnitudes of sets task is somewhat similar to symbolic magnitude judgment task used in the current study]. The TEMA is a known predictor of children’s math ability (Halberda et al., 2008; Mazzocco et al., 2011).

#### Addition Problem-Solving Tasks

The Time 2 single-digit addition (SDA) task comprised 16 single digit addition problems (2 + 4, 2 + 5, 2 + 6, 2 + 7, 3 + 5, 3 + 6, 3 + 8, 4 + 2, 5 + 2, 5 + 3, 5 + 7, 6 + 2, 6 + 3, 7 + 2, 7 + 5, 8 + 3); the Time 3 mixed SDA and double-digit addition problems (SDA/DDA) task comprised 12 mental addition problems (e.g., 8 + 13) in which the sum of the addends was less than 50; and the Time 4 double-digit addition (DDA) task comprised 24 mental addition problems (e.g., 35 + 47) in which the sum of the addends was less than 100. For all tasks, children received practice trials to familiarize them with task requirement (to solve problems as quickly and as accurately as possible). All problems were presented in a random order, and problems appeared in the center of the screen, in the form of a + b = [Note, no identical single digits (e.g., 2 + 2, 4 + 34, 33 + 43) were included in problem sets.]. Problems correctness and problem-solving RTs were recorded.

### Analytic Approach

An initial model examined whether addition RTs were predictive of each other over time, and whether TEMA-2 measured at Time 1 was a predictor of addition RTs assessed at Time 2, Time 3, and Time 4. A second model examined the relationships between dot enumeration profiles, magnitude comparison RTs and TEMA and VSWM measured at Time 1, to assess whether these measures predicted addition RTs at Time 2, Time 3, and Time 4 respectively. Since NVIQ was collected at Time 3, it was only included in the model to predict Time 3 and Time 4 addition RTs. [Note, we included gender in our analyses since it has been found to be associated with math ability (Carr and Alexeev, 2011; Paul and Reeve, 2016); however, we do not make specific predictions about gender.]

All model parameters were calculated using full-information maximum likelihood estimation, which provides robust estimates in the case of missing data (n_T1_ = 276; n_T2_ = 247; n_T3_ = 201; n_T4_ = 176). Analyses were conducted using MPlus version 7 (Muthén et al., 1998–2013). Model fit was evaluated using three types of indices: (1) relative chi-square (normed chi-square), which is less sensitive to sample size (χ^2^/df < 2) (Ullman, 2001); (2) relative indices [Comparative Fit Index (CFI), Tucker-Lewis Index (TLI)] (Kline, 2005); and (3) absolute fit indices [standardized root mean square residual (SRMR), root mean square error of approximation (RMSEA)] (e.g., Hu and Bentler, 1999; MacCallum and Austin, 2000). Standard benchmark values were used to define acceptable model fit (CFI/TLI > 0.90, SRMR/RMSEA < 0.10) and good model fit (CFI/TLI > 0.95, SRMR/RMSEA < 0.08) (Marsh et al., 2004).

## Results

### Descriptive Statistics

The means and SDs for all measures and correlations among them are reported in **Table 1**. Children had a VSWM span of between 3 and 4 items, which is consistent with age-norms (Farrell Pagulayan et al., 2006). NVIQ was similar to previously Australian findings (Cotton et al., 2005). As expected, addition problem solving RTs increased over time, as problem difficulty increased. Significant correlations were found between dot enumeration profile and TEMA-2, VSWM, and addition RT over time; however, magnitude comparison RTs were not correlated with other measures. NVIQ was significantly correlated with VSWM, the TEMA, dot enumeration profile membership and addition RTs at Time 3 and Time 4. The TEMA and the three addition RTs were significantly inter-correlated over time. Accuracy for the magnitude comparison task was quite high (*M* = 0.94, *SD* = 0.05), and near ceiling for most children. Accuracy for SDA (*M* = 0.89, *SD* = 0.07), SDA/DDA (*M* = 0.87, *SD* = 0.03), and DDA (*M* = 0.85, *SD* = 0.10) tasks was also high.

**Table 1:** Bivariate correlations (bias-corrected bootstrap estimates, 95% confidence intervals) between cognitive, core number, and mental addition response times.

Variable	1	2	3	4	5	6	7	8	9
(1) SDA (T2)	1								
	–								
(2) SDA/DDA (T3)	0.48^∗∗∗^	1							
	[0.37, 0.58]	–							
(3) DDA (T4)	0.40^∗∗∗^	0.57^∗∗∗^	1						
	[0.27, 0.52]	[0.44, 0.67]	–						
(4) DE-S3 (T1)	-0.27^∗∗∗^	-0.31^∗∗∗^	-0.34^∗∗∗^	1					
	[-0.36, -0.15]	[-0.42, -0.18]	[-0.45, -0.24]	–					
(5) DE-S2 (T1)	0.13^∗^	0.14^∗^	0.17^∗^	-0.74^∗∗∗^	1				
	[0.01, 0.24]	[0.00, 0.27]	[0.03, 0.32]	[-0.81, -0.68]	–				
(6) TEMA (T1)	-0.51^∗∗∗^	-0.43^∗∗∗^	-0.33^∗∗∗^	0.31^∗∗∗^	-0.15^∗∗^	1			
	[-0.58, -0.44]	[-0.53, -0.31]	[-0.46, -0.18]	[0.20, 0.41]	[-0.28, -0.04]	–			
(7) VSWM (T1)	-0.20^∗∗^	-0.21^∗∗^	-0.26^∗∗∗^	0.21^∗∗∗^	-0.13^∗^	0.31^∗∗∗^	1		
	[-0.32, -0.08]	[-0.34, -0.08]	[-0.40, -0.11]	[0.10, 0.33]	[-0.25, -0.02]	[0.20, 0.42]	–		
(8) MC (T1)	0.12	0.09	0.07	-0.05	0.05	-0.10	-0.06	1	
	[-0.02, 0.26]	[-0.04, 0.23]	[-0.07, 0.20]	[-0.15, 0.06]	[-0.07, 0.16]	[-0.22, 0.03]	[-0.17, 0.06]	–	
(9) NVIQ (T3)	-0.25^∗∗∗^	-0.35^∗∗∗^	-0.24^∗∗^	0.21^∗∗^	-0.11	0.37^∗∗∗^	0.25^∗∗∗^	-0.01	1
	[-0.39, -0.11]	[-0.47, -0.22]	[-0.36, -0.08]	[0.09, 0.35]	[-0.25, 0.03]	[0.25, 0.50]	[0.12, 0.38]	[-0.14, 0.12]	–
Mean	5.89	7.42	7.59	0.35	0.51	105.43	3.68	1.82	29.47
*SD*	2.78	3.41	3.53	0.48	0.50	13.27	0.67	0.52	4.46


*SDA, single-digit addition; DDA, double-digit addition; DE-S3, Membership in Fast Dot Enumeration Profile, relative to Slow Dot Enumeration Profile membership; DE-S2, Membership in Moderate Dot Enumeration Profile, relative to Slow Dot Enumeration Profile membership; TEMA, Test of Early Mathematics Achievement; MC, magnitude comparison; VSWM, visuo-spatial working memory span; NVIQ, non-verbal IQ. Product-moment correlations were estimated between continuous variables, while biseral correlations were estimated between continuous and dichotomous variables. ^∗∗∗^*p* < 0.001, ^∗∗^*p* < 0.01, ^∗^*p* < 0.05.*

### Associations between TEMA-2 and Mental Addition RTs Over Time

**FIGURE 1:**
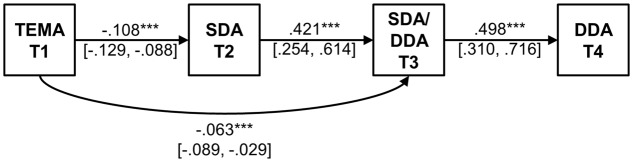
Path diagram for model showing the relationship between single-digit addition (SDA-T2), single-digit/double digit addition task (SDA/DDA-T3) and double-digit addition (DDA-T4), predicted by TEMA-2 scores measured at Time 1 (T1). Unstandardized path weights are shown (with bias-corrected bootstrap standard errors) for significant paths. SDA, single-digit addition; DDA, double-digit addition; TEMA, Test of Early Mathematics Achievement. ^∗∗∗^*p* < 0.001.

The significant paths and associated unstandardized path weights (with bias-corrected bootstrap standard errors) between TEMA-2, SDA, SDA/DDA, and DDA (Model 1) are presented in **Figure 1**. For a one point increase in TEMA test scores at Time 1, children’s addition problem-solving response times were predicted to be 0.11 s faster at Time 2, and 0.06 s faster at Time 3. For a 1 s increase in addition problem-solving response times at Time 2, children’s addition problem-solving response times were predicted to be 0.42 s faster at Time 3. For a 1 s increase in addition problem-solving response times at Time 3, children’s addition problem-solving response times were predicted to be 0.50 s faster at Time 4. As Model 1 is fully-specified no fit statistics are reported.

#### Association between Core Number, VSWM, NVIQ and Mental Addition RTs Over Time

**FIGURE 2:**
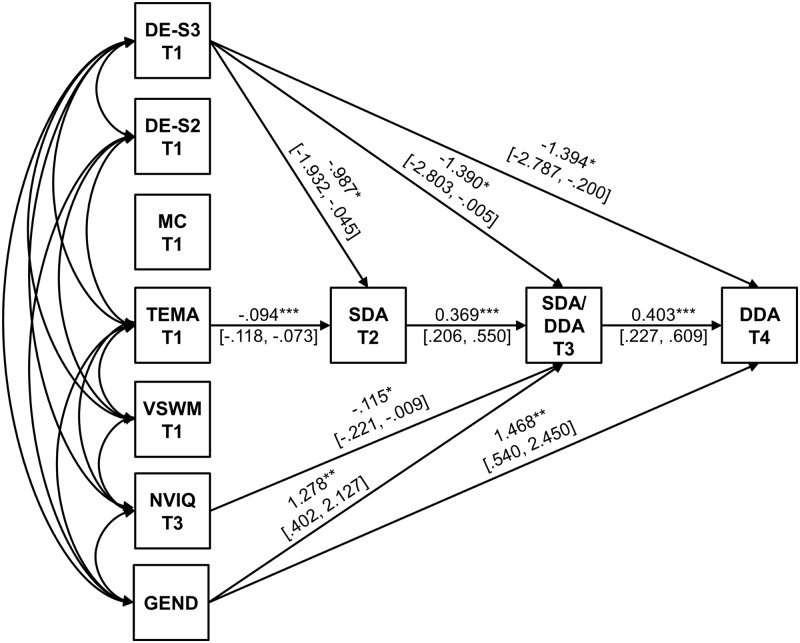
Path diagram for Model 2 showing the relationship between SDA tasks at T2, SDA/DDA at T3 and DDA at T4, predicted by TEMA, DE profile membership measured at T1, gender and NVIQ scores at T3. Correlations between measures are reported in text. Unstandardized path weights are shown (with bias-correct bootstrap standard errors) for significant paths. SDA, single-digit addition; DDA, double-digit addition; DE-S3, Membership in Fast Dot Enumeration Profile, relative to Slow Dot Enumeration Profile membership; DE-S2, Membership in Moderate Dot Enumeration Profile, relative to Slow Dot Enumeration Profile membership; TEMA, Test of Early Mathematics Achievement; MC, magnitude comparison; VSWM, visuo-spatial working memory span; NVIQ, non-verbal IQ. ^∗∗∗^*p* < 0.001, ^∗∗^*p* < 0.01, ^∗^*p* < 0.05.

The model provided a good fit to the data, χ^2^(1) = 0.702, *p* = 0.402; normed chi-square (χ^2^/df) = 0.70, RMSEA = 0.00 [90% CI = 0.00, 0.15], CFI/TLI = 1.00/1.00, SRMR = 0.007. Correlations between Time 1 covariates are presented in **Table 2**. All significant paths and associated unstandardized path weights (and bias-corrected bootstrap standard errors), are shown in **Figure 2**.

**Table 2:** Path model correlations (bias-corrected bootstrap estimates, 95% confidence intervals) between Time 1 covariates.

Variable	1	2	3	4	5	6	7
(1) DE-S3 (T1)	1						
	–						
(2) DE-S2 (T1)	-0.74^∗∗∗^	1					
	[-0.81, -0.68]	–					
(3) TEMA (T1)	0.31^∗∗∗^	-0.15^∗^	1				
	[0.20, 0.41]	[-0.27, -0.04]	–				
(4) MC (T1)	-0.05	0.05	-0.10	1			
	[-0.16, 0.07]	[-0.07, 0.16]	[-0.22, 0.02]	–			
(5) VSWM (T1)	0.21^∗∗∗^	-0.13^∗^	0.31^∗∗∗^	-0.06	1		
	[0.09, 0.33]	[-0.24, -0.01]	[0.19, 0.42]	[-0.17, 0.06]	–		
(6) Gender	-0.16^∗∗^	0.12^∗^	-0.12^∗^	0.02	0.01	1	
	[-0.28, -0.04]	[0.00, 0.24]	[-0.25, 0.02]	[-0.09, 0.15]	[-0.11, 0.13]	–	
(7) NVIQ (T3)	0.21^∗∗^	-0.12	0.37^∗∗∗^	0.00	0.24^∗∗∗^	-0.20^∗∗^	1
	[0.08, 0.33]	[-0.25, 0.02]	[0.25, 0.49]	[-0.13, 0.13]	[0.11, 0.37]	[-0.32, -0.05]	–


*DE-S3, Membership in Fast Dot Enumeration Profile, relative to Slow Dot Enumeration Profile membership; DE-S2, Membership in Moderate Dot Enumeration Profile, relative to Slow Dot Enumeration Profile membership; TEMA, Test of Early Mathematics Achievement; MC, magnitude comparison; VSWM, visuo -spatial working memory span; NVIQ, non-verbal IQ. Biseral correlations were estimated between continuous and dichotomous variables, while tetrachoric correlations were estimated between dichotomous and dichotomous variables. ^∗∗∗^*p* < 0.001, ^∗∗^*p* < 0.01, ^∗^*p* < 0.05.*

Higher TEMA-2 scores were associated with membership in the fast dot enumeration profiles, higher VSWM and NVIQ. Lower TEMA-2 scores were associated with medium dot enumeration profile membership, and for girls relative to boys. Higher NVIQ was associated with higher VSWM and membership of the fast dot enumeration profile, while lower NVIQ was associated with girls relative to boys. Girls were less likely to be in the fast dot enumeration profile, relative to boys, but more likely to be in the medium dot enumeration profile. Higher VSWM was associated with membership in the fast dot enumeration profile.

Similar to the first model, a one point increase in TEMA scores predicted 0.09 s faster single-digit addition problem-solving RTs (Time 2). A 1 s increase in single-digit addition problem-solving RTs at Time 2 predicted 0.37 s slower single-digit/double-digit addition problem-solving RTs at Time 3, while a 1 s increase single-digit/double-digit addition problem-solving RTs at Time 3 predicted 0.40 s slower double-digit addition problem-solving RTs at Time 4. These findings suggest the inclusion of covariates in the model did not alter the relationship between addition problem-solving RTs over time. Dot enumeration profile membership predicted addition problem-solving RTs at Time 2, Time 3, and Time 4. Membership in the fast dot enumeration profile, compared to the slow dot enumeration profile, at Time 1 predicted a 0.99 s faster single-digit addition problem-solving RTs at Time 2, 1.39 s faster single-digit/double-digit addition RTs at Time 3, and 1.39 s faster double-digit addition RTs at Time 4, respectively. For one point increase in NVIQ at Time 3, single-digit/double-digit addition problem-solving RTs were 0.12 s faster at Time 3. Girls were 1.28 and 1.47 s slower, than boys, at solving addition problems at Time 3 and Time 4, respectively. Performance on the magnitude comparison task and VSWM did not predict unique variance to the path model: neither measure predicted addition problem-solving RTs at any time point.

## Discussion

The study investigated the degree to which the TEMA, dot enumeration, magnitude comparison abilities, taking into account by VSWM assessed at school entry, and NVIQ assessed 3 years later, predicted different mental addition problem solving test RTs 2, 4, and 6 years after initial testing. Four findings are of note. First, the TEMA, assessed at 5-years (Time 1), predicted addition RTs at 6–7 years (Time 2), but not 8–9 (Time 3) or 10–11 years (Time 4). Second, addition RTs at one time point predicted addition RTs at the subsequent point, even though the addition tasks were different, with the size of the effect increasing over time. This finding highlights the componential nature of math ability development. Third, dot enumeration profile membership, but not magnitude comparison RT, was a reliable predictor of addition RTs over time. More specifically, dot enumeration profile membership at Time 1 predicted addition RTs at Time 2, Time 3, and Time 4. Fourth, while NVIQ predicted addition RTs at Time 3, it was unrelated to addition RTs at Time 4. Girls had relatively slower addition problem-solving RTs, than boys, at Time 3 and Time 5, but not at Time 2. Neither magnitude comparison RTs nor VSWM predicted addition RTs at any time point. In sum, the pattern of findings show the TEMA predicted mental addition abilities in the short term, and dot enumeration profile membership predicted mental addition problem solving RTs in both the short and long term. Moreover, dot enumeration profile membership and TEMA scores were partially independent of each other and appear to reflect different math competencies.

The findings suggest the TEMA, assessed at school entry, is a useful predictor of mental addition RTs, and ipso facto math problem solving ability, in the relatively short term; however, its value lessens over time in predicting more complex mental addition problem solving RTs. While the predictive value of the TEMA decreased over time, the predictive value of dot enumeration increased over time. The dot enumeration profiles, assessed at school entry, not only predicted addition problem solving RTs in the short and long term, it made an independent contribution to the prediction model to the TEMA. Some measures did not contribute to the model predicting mental addition RTs over time. Magnitude comparison RT, a well-established core number index, was not correlated with other measures. This was surprising since magnitude comparison and the mental addition were based on RT measures. It is possible the number comparison task measure may be insensitive in 5-year-olds (see Reeve et al., 2012). And while VSWM was correlated with other measures, it did not contribute to the prediction model. As noted below, it is possible dot enumeration profile membership is a proxy measure of VSWM. NVIQ was related to the Time 3 SDA/DDA RT, but not the Time 4 DDA RT. It is possible the demands of the DDA task required other cognitive resources. Gray and Reeve (2016), for example, argue complex math problems likely require cognitive switching and inhibition strategies, which tend to be only moderately correlated with general intelligence measures.

Although performances on the three mental addition tasks were correlated, highlighting the componential nature of math development, the finding that the TEMA was unrelated to double digit addition requires comment. One possibility is the TEMA is more related to emerging math abilities, compared to more complex or cognitively demanding math abilities (see Chu et al., 2016 for a similar argument). This suggestion is consistent with those who have argued that magnitude representation abilities are more important for emerging math abilities (e.g., number fact knowledge) but not more complex abilities (Geary et al., 2012). However, these suggestions do not account for the independent predictive significance of dot enumeration abilities.

The pattern of findings suggest the TEMA and dot enumerations measures together are useful measures of young children’s math learning potential and, ipso facto, could serve as a basis for designing a math intervention for children at risk. However, caution should be exercised in accepting this possibility uncritically. While the math components of the TEMA are well-known and could provide a partial basis of an intervention model (Mazzocco and Thompson, 2005; Mazzocco et al., 2013), the conceptual basis of dot enumeration abilities is less clear and needs to be better articulated.

The dot enumeration task was designed to assess claims about the importance of individual differences in precise number abilities for the development of numerical cognition. Research has confirmed that individual differences in this core number ability are not only relatively stable over time, they are consistently associated with children’s math problem solving abilities (Reeve et al., 2012; Gray and Reeve, 2014). In the Reeve et al. research, dot enumeration abilities were examined as a function of four parameters, described earlier, and analyzed using latent class modeling. This approach yielded three distinct dot enumeration profiles each of which differed from each other in subitizing range, subitizing and count RT slopes, and subitizing intercept. Of particular interest are the children who were assigned to the “slow” profile – they were not only slower than children assigned to the “medium” and “fast” profiles, their subitizing range was more limited. More specifically, children in the “slow” profile appeared to count individual dots in answer to question of the “how many dots,” rather than subitizing *n* ≤ 4 dots in a relatively automatic manner. In sum, the “slow” dot enumeration profile is distinguishable from the other profiles in terms of response speed and subitizing range, both of which could have implications for math assessment and intervention.

It is possible dot enumeration response speed reflects general processing speed and, by extension, a general cognitive factor. This possibility reflects the view that processing speed is a proxy measure of intelligence (Coyle et al., 2011; however, see Cepeda et al., 2013). Findings in support of a general processing speed argument from the present study are mixed. On the one hand, a moderate negative association was found between dot enumeration profile and mental addition RTs, and on the other, no association was found between magnitude comparison RTs and addition RTs. It is evident the general responses speed argument is complex and may involve different cognitive functions and affect cognitive domains differently (see Cepeda et al., 2013). Nevertheless, insofar as a general processing speed limitation argument is plausible, it is unlikely response speed training would affect dot enumeration efficiency.

The three dot enumeration profiles also differed in their subitizing ranges. Some children in the “slow” profile had very small subitizing ranges, while some children in the “fast” profile had adult-like ranges (*n* ≥ 4). It could be argued subitizing ranges reflects a working memory capacity. We suggest, however, this explanation needs to be treated with caution for at least two reasons. First, the correlation between VSWM and subitizing range is relatively small (0.21 in the current data). Second, some individuals with dyscalculia are unable to subitize and count items one-by-one instead, but nevertheless possess a good working memory (Butterworth, 1999).

We suggest that children with a limited subitizing range, as is evident in the slow profile, may lack the ability to readily extract pattern or grouping information from small sets of dots (Butterworth, 2003; Ashkenazi et al., 2013). Why might this be important for numerical cognition? The ability to “know” the number “2” or “3” can be represented by a collection of two or three dots respectively, without counting individual dots, is an index of set knowledge (Butterworth, 2010); and set manipulation represents an important aspect of the development of numerical cognition (Gallistel and Gelman, 1992). The degree to which set knowledge changes in childhood is yet to be specified precisely; however, in the absence of set knowledge, numerical reasoning is likely to be difficult, as is evident in individuals with developmental dyscalculia, who appear to lack the ability to extract information from small sets of dots at a glance (Butterworth, 2010). Indeed, Laurillard (2016) has developed “Numberbeads” math software designed to facilitate children’s set knowledge and overcome difficulties indicated by poor dot enumeration abilities. Along with Laurillard, we suggest a math intervention based on improving set knowledge would be a fruitful avenue to investigate for educators (see also Butterworth et al., 2011).

### Limitations and Future Research

There are at least two broad potential limitations of the current study could to be addressed in future research, and which may have implications for a math interventions. First, the data for the present study were collected at school entry. It is possible that this is not the optimal time to collect reliable data. A number of factors affect measurement reliability (e.g., familiarity with assessment, task difficulty), and assessing math learning potential later in the school year or at the beginning of the next school year might yield more reliable data (Schmitt et al., 2017). Although the TEMA was associated with mental addition RTs when assessed concurrently at school entry, its predictive value diminished over time. Later or repeat testing of the TEMA might yield more reliable math ability information. In the present study we used the standardized TEMA test score. Some have found an analysis of the subscale measures of the TEMA provide information about older children’s conceptual and procedural math understanding (Ryoo et al., 2015). It is an open question whether a subscale analysis of younger children performance would similarly be useful. Finally, we acknowledge we used the earlier TEMA-2, rather than the current TEMA-3, in our research. We do not consider this decision materially affected our findings.

A second potential limitation is we focused on mental addition RT as the to-be-predicted measure. We are confident RT is a proxy measure for single digit addition strategic ability since there is a close relationship between the conceptual sophistication of SDA strategy-use and the speed taken to execute different strategies (see Canobi et al., 1998, 2002; Paul and Reeve, 2016). However, we are less confident the RT measures of the SDA/DDA and DDA tasks similarly reflected strategic reasoning activity, simply because, as far as we can ascertain, no one has conducted the appropriate problem-solving strategy analysis for these tasks. Although we focused on mental addition in the pre-adolescent years because of its importance in the math curriculum, we recognize the pattern of findings may differ for other math measures (e.g., subtraction, multiplication, division) that might depend on different competencies.

Although gender differences were not the focus of our study, we, like others (Paul and Reeve, 2016), found differences in a performance measure (see Carr and Alexeev, 2011). In the present study boys solved problems slightly faster than girls; however, no gender differences were observed in problem solving accuracy. We suggest future research investigate the reasons for these speed differences.

## Conclusion

The results of the present study showed a standardized measure of mathematics achievement (TEMA) predicted mental addition RTs in the short term, but not the long term, while dot enumeration profile membership predicted mental addition RTs in the short and long term. Moreover, dot enumeration profile membership and TEMA measures were partially independent of each other in predicting mental addition RTs over time. We suggest that these two measures together could be used as a screening device to assess at risk children of math delay. While dot enumeration profile membership was the more powerful predictor of the two measures, it is evident research is required to unpack the conceptual basis of the dot enumeration measure. More specifically, it was suggested the subitizing component of dot enumeration reflect set based reasoning abilities, regarded as critically important for the development of numerical abilities. On the basis of this suggestion, we contend intervention strategies should focus on facilitating set based reasoning via dot enumeration tasks.

## Ethics Statement

This study was carried out in accordance with the recommendations of the Human Research Ethics Committee of the University of Melbourne. Written informed consent was obtained from the parents of children who participated in the study. All subjects gave written informed consent in accordance with the Declaration of Helsinki. The protocol was approved by the Human Research Ethics Committee of the University of Melbourne.

## Author Contributions

All authors listed have made a substantial, direct and intellectual contribution to the work, and approved it for publication.

## Conflict of Interest Statement

The authors declare that the research was conducted in the absence of any commercial or financial relationships that could be construed as a potential conflict of interest.

## References

[B1] Ashkenazi S. Mark-Zigdon N. Henik A. (2013). Do subitizing deficits in developmental dyscalculia involve pattern recognition weakness? *Dev. Sci.* 16 35–46. 10.1111/j.1467-7687.2012.01190.x 23278925

[B2] Australian Curriculum Assessment and Reporting Authority (2017). Available at: https://www.australiancurriculum.edu.au/f-10-curriculum/general-capabilities/numeracy/

[B3] Barrouillet P. Lépine R. (2005). Working memory and children’s use of retrieval to solve addition problems. *J. Exp. Child Psychol.* 91 183–204. 10.1016/j.jecp.2005.03.002 15925643

[B4] Bartelet D. Vaessen A. Blomert L. Ansari D. (2014). What basic number processing measures in kindergarten explain unique variability in first-grade arithmetic proficiency? *J. Exp. Child Psychol.* 117 12–28. 10.1016/j.jecp.2013.08.010 24128690

[B5] Bull R. Espy K. A. Wiebe S. A. (2008). Short-term memory, working memory, and executive functioning in preschoolers: longitudinal predictors of mathematical achievement at age 7 years. *Dev. Neuropsychol.* 33 205–228. 10.1080/87565640801982312 18473197 PMC2729141

[B6] Butterworth B. (1999). *The Mathematical Brain.* London, UK: Macmillan.

[B7] Butterworth B. (2003). *Dyscalculia Screener.* London: nferNelson Publishing Company Ltd.

[B8] Butterworth B. (2005). The development of arithmetical abilities. *J. Psychol. Psychiatry* 46 3–18. 10.1111/j.1469.7610.2004.00374.x 15660640

[B9] Butterworth B. (2010). Foundational numerical capacities and the origins of dyscalculia. *Trends Cogn. Sci.* 14 534–541. 10.1016/j.tics.2010.09.007 20971676

[B10] Butterworth B. Varma S. Laurillard D. (2011). Dyscalculia: from brain to education. *Science (New York, N.Y.)* 332 1049–1053. 10.1126/science.1201536 21617068

[B11] Canobi K. H. Reeve R. A. Pattison P. E. (1998). The role of conceptual understanding in children’s addition problem solving. *Dev. Psychol.* 34 882–891. 10.1037//0012-1649.34.5.882 9779735

[B12] Canobi K. H. Reeve R. A. Pattison P. E. (2002). Young children’s understanding of addition concepts. *Educ. Psychol.* 22 513–532. 10.1080/0144341022000023608

[B13] Carr M. Alexeev N. (2011). Fluency, accuracy, and gender predict developmental trajectories of arithmetic strategies. *J. Educ. Psychol.* 103 617–631. 10.1037/a0023864

[B14] Cepeda N. J. Blackwell K. A. Munakata Y. (2013). Speed isn’t everything: complex processing speed measures mask individual differences and developmental changes in executive control. *Dev. Sci.* 16 269–286. 10.1111/desc.12024 23432836 PMC3582037

[B15] Chu F. W. vanMarle K. Geary D. C. (2016). Predicting Children’s reading and mathematics achievement from early quantitative knowledge and domain-general cognitive abilities. *Front. Psychol.* 7:775. 10.3389/fpsyg.2016.00775 27252675 PMC4879431

[B16] Cotton S. M. Kiely P. M. Crewther D. P. Thomson B. Laycock R. Crewther S. G. (2005). A normative and reliability study for the Raven’s coloured progressive matrices for primary school aged children from Victoria, Australia. *Pers. Individ. Differ.* 39 647–659. 10.1016/j.paid.2005.02.015

[B17] Coyle T. R. Pillow D. R. Snyder A. C. Kochunov P. (2011). Processing speed mediates the development of general intelligence (g) in adolescence. *Psychol. Sci.* 22 1265–1269. 10.1177/0956797611418243 21931154 PMC3725745

[B18] de Paula J. J. Malloy-Diniz L. F. Romano-Silva M. A. (2016). Reliability of working memory assessment in neurocognitive disorders: a study of the digit span and corsi block-tapping tasks. *Rev. Bras. Psiquiatr.* 38 262–263. 10.1590/1516-4446-2015-1879 27579598 PMC7194262

[B19] De Smedt B. Verschaffel L. Ghesquière P. (2009). The predictive value of numerical magnitude comparison for individual differences in mathematics achievement. *J. Exp. Child Psychol.* 103 469–479. 10.1016/j.jecp.2009.01.010 19285682

[B20] Desoete A. Ceulemans A. De Weerdt F. Pieters S. (2012). Can we predict mathematical learning disabilities from symbolic and non-symbolic comparison tasks in kindergarten? Findings from a longitudinal study. *Br. J. Educ. Psychol.* 82(Pt 1), 64–81. 10.1348/2044-8279.002002 21199482

[B21] Donlan C. Baroody A. J. Dowker A. (2003). “The early numeracy of children with specific language impairments,” in *The Development of Arithmetic Concepts and Skills: Constructive Adaptive Expertise*, eds Baroody A. Dowker A. (Mahwah, NJ: Lawrence Erlbaum Associates), 337–358.

[B22] Farrell Pagulayan K. Busch R. M. Medina K. L. Bartok J. A. Krikorian R. (2006). Developmental normative data for the Corsi Block-tapping task. *J. Clin. Exp. Neuropsychol.* 28 1043–1052. 10.1080/13803390500350977 16822742

[B23] Feigenson L. Dehaene S. Spelke E. (2004). Core systems of number. *Trends Cogn. Sci.* 8 307–314. 10.1016/j.tics.2004.05.002 15242690

[B24] Feigenson L. Libertus M. E. Halberda J. (2013). Links between the intuitive sense of number and formal mathematics ability. *Child Dev. Perspect.* 7 74–79. 10.1111/cdep.12019 24443651 PMC3891767

[B25] Friso-van den Bos I. van der Ven S. H. Kroesbergen E. H. van Luit J. E. (2013). Working memory and mathematics in primary school children: a meta-analysis. *Educ. Res. Rev.* 10 29–44. 10.1016/j.edurev.2013.05.003

[B26] Gallistel C. R. Gelman R. (1992). Preverbal and verbal counting and computation. *Cognition* 44 43–74. 10.1016/0010-0277(92)90050-R 1511586

[B27] Geary D. C. Hoard M. K. Byrd-Craven J. DeSoto M. C. (2004). Strategy choices in simple and complex addition: contributions of working memory and counting knowledge for children with mathematical disability. *J. Exp. Child Psychol.* 88 121–151. 10.1016/j.jecp.2004.03.002 15157755

[B28] Geary D. C. Hoard M. K. Nugent L. Bailey D. H. (2012). Mathematical cognition deficits in children with learning disabilities and persistent low achievement: a five-year prospective study. *J. Educ. Psychol.* 104 206–223. 10.1037/a0025398 27158154 PMC4855881

[B29] Geary D. C. Hoard M. K. Nugent L. Bailey D. H. (2013). Adolescents’ functional numeracy is predicted by their school entry number system knowledge. *PLOS ONE* 8:e54651. 10.1371/journal.pone.0054651 23382934 PMC3559782

[B30] Geary D. C. Hoard M. K. Nugent L. Byrd-Craven J. (2007). “Strategy use, long-term memory, and working memory capacity,” in *Why Is Math So Hard for Some Children? The Nature and Origins of Mathematical Learning Difficulties and Disabilities*, eds Berch D. E. Mazzocco M. M. (Baltimore, MD: Brookes Publishing Company), 83–105.

[B31] Geary D. C. Nicholas A. Li Y. Sun J. (2017). Developmental change in the influence of domain-general abilities and domain-specific knowledge on mathematics achievement: an eight-year longitudinal study. *J. Educ. Psychol.* 109 680–693. 10.1037/edu0000159 28781382 PMC5542417

[B32] Ginsburg H. P. Baroody A. (2003). *Test of Early Mathematics Ability*, 3rd Edn Austin, TX: Pro-Ed.

[B33] Ginsburg H. P. Baroody A. J. (1990). *Test of Early Mathematics Ability*, 2nd Edn Austin, TX: Pro-Ed.

[B34] Gray S. A. Reeve R. A. (2014). Preschoolers’ dot enumeration abilities are markers of their arithmetic competence. *PLOS ONE* 9:e94428. 10.1371/journal.pone.0094428 24714052 PMC3979837

[B35] Gray S. A. Reeve R. A. (2016). Number-specific and general cognitive markers of preschoolers’ math ability profiles. *J. Exp. Child Psychol.* 147 1–21. 10.1016/j.jecp.2016.02.004 26985575

[B36] Halberda J. Mazzocco M. M. Feigenson L. (2008). Individual differences in non-verbal number acuity correlate with maths achievement. *Nature* 455 665–668. 10.1038/nature07246 18776888

[B37] Holmes J. Adams J. W. Hamilton C. J. (2008). The relationship between visuospatial sketchpad capacity and children’s mathematical skills. *Eur. J. Cogn. Psychol.* 20 272–289. 10.1080/09541440701612702

[B38] Hornung C. Schiltz C. Brunner M. Martin R. (2014). Predicting first-grade mathematics achievement: the contributions of domain-general cognitive abilities, nonverbal number sense, and early number competence. *Front. Psychol.* 5:272. 10.3389/fpsyg.2014.00272 24772098 PMC3983481

[B39] Hu L. T. Bentler P. M. (1999). ”Cutoff criteria for fit indexes in covariance structure analysis: conventional criteria versus new alternatives. *Struct. Equat. Model.* 6 1–55. 10.1080/10705519909540118

[B40] Hyde D. C. Spelke E. S. (2011). Neural signatures of number processing in human infants: evidence for two core systems underlying numerical cognition. *Dev. Sci.* 14 360–371. 10.1111/j.1467-7687.2010.00987.x 21399717 PMC3050652

[B41] Imbo I. Vandierendonck A. (2007). The development of strategy use in elementary school children: working memory and individual differences. *J. Exp. Child Psychol.* 96 284–309. 10.1016/j.jecp.2006.09.001 17046017

[B42] Kable J. A. Taddeo E. Strickland D. Cole C. D. (2015). Community translation of the math interactive learning experience program for children with FASD. *Res. Dev. Disabil.* 39 1–11. 10.1016/j.ridd.2014.12.031 25601483

[B43] Kessels R. P. van Zandvoort M. J. Postma A. Kappelle L. J. de Haan E. H. (2000). The corsi block-tapping task: standardization and normative data. *Appl. Neuropsychol.* 7 252–258. 10.1207/S15324826AN0704_8 11296689

[B44] Kline R. B. (2005). *Principles and Practice of Structural Equation Modeling*, 2nd Edn New York, NY: The Guilford Press.

[B45] Lan X. Legare C. H. Ponitz C. C. Li S. Morrison F. J. (2011). Investigating the links between the subcomponents of executive function and academic achievement: a cross-cultural analysis of Chinese and American preschoolers. *J. Exp. Child Psychol.* 108 677–692. 10.1016/j.jecp.2010.11.001 21238977

[B46] Laurillard D. (2016). Learning number sense through digital games with intrinsic feedback. *Australas. J. Educ. Technol.* 32 32–44. 10.14742/ajet.3116

[B47] Lee K. Bull R. (2016). Developmental changes in working memory, updating, and math achievement. *J. Educ. Psychol.* 108 869–882. 10.1037/edu0000090

[B48] MacCallum R. C. Austin J. T. (2000). Applications of structural equation modeling in psychological research. *Annu. Rev. Psychol.* 51:201 10.1146/annurev.psych.51.1.201 10751970

[B49] Marsh H. W. Hau K.-T. Wen Z. (2004). In search of golden rules: comment on hypothesis testing approaches to setting cutoff values for fit indexes and dangers in overgeneralizing Hu & Bentler’s (1999) findings. *Struct. Equat. Model.* 11 320–341. 10.1207/s15328007sem1103_2

[B50] Mazzocco M. M. Feigenson L. Halberda J. (2011). Preschoolers’ precision of the approximate number system predicts later school mathematics performance. *PLOS ONE* 6:e23749. 10.1371/journal.pone.0023749 21935362 PMC3173357

[B51] Mazzocco M. M. Murphy M. M. Brown E. C. Rinne L. Herold K. H. (2013). Persistent consequences of atypical early number concepts. *Front. Psychol.* 4:486. 10.3389/fpsyg.2013.00486 24027540 PMC3761157

[B52] Mazzocco M. M. Myers G. F. (2003). Complexities in identifying and defining mathematics learning disability in the primary school-age years. *Ann. Dyslexia* 53 218–253. 10.1007/s11881-003-0011-7 19750132 PMC2742419

[B53] Mazzocco M. M. Thompson R. E. (2005). Kindergarten predictors of math learning disability. *Learn. Disabil. Res. Pract.* 20 142–155. 10.1111/j.15405826.2005.00129.x 20084182 PMC2806680

[B54] Meyer M. Salimpoor V. Wu S. Geary D. Menon V. (2010). Differential contribution of specific working memory components to mathematics achievement in 2nd and 3rd graders. *Learn. Individ. Differ.* 20 101–109. 10.1016/j.lindif.2009.08.004 21660238 PMC3109434

[B55] Milner B. (1971). Interhemispheric differences in the localization of psychological processes in man. *Br. Med. Bull.* 27 272–277. 10.1093/oxfordjournals.bmb.a070866 4937273

[B56] Muthén B. (2003). Statistical and substantive checking in growth mixture modeling: comment on Bauer and Curran (2003). *Psychol. Methods* 8 369–377. 10.1037/1082-989X.8.3.369 14596497

[B57] Muthén L. K. Muthén B. O. (1998–2013). *Mplus User’s Guide*, Vol. 7 Los Angeles, CA: Muthén & Muthén

[B58] Passolunghi M. C. Siegel L. S. (2004). Working memory and access to numerical information in children with disability in mathematics. *J. Exp. Child Psychol.* 88 348–367. 10.1016/j.jecp.2004.04.002 15265681

[B59] Paul J. M. Reeve R. A. (2016). Relationship between single digit addition strategies and working memory reflects general reasoning sophistication. *Learn. Instr.* 42 113–122. 10.1016/j.learninstruc.2016.01.011

[B60] Peng P. Namkung J. Barnes M. Sun C. (2016). A meta-analysis of mathematics and working memory: moderating effects of working memory domain, type of mathematics skill, and sample characteristics. *J. Educ. Psychol.* 108 455–473. 10.1037/edu0000079

[B61] Purpura D. J. Ganley C. M. (2014). Working memory and language: skill-specific or domain-general relations to mathematics? *J. Exp. Child Psychol.* 122 104–121. 10.1016/j.jecp.2013.12.009 24549230

[B62] Purpura D. J. Reid E. E. Eiland M. D. Baroody A. J. (2015). Using a brief preschool early numeracy skills screener to identify young children with mathematics difficulties. *Sch. Psychol. Rev.* 44 41–59. 10.17105/SPR44-1.41-59

[B63] Purpura D. J. Schmitt S. A. Ganley C. M. (2017). Foundations of mathematics and literacy: the role of executive functioning components. *J. Exp. Child Psychol.* 153 15–34. 10.1016/j.jecp.2016.08.010 27676183

[B64] Raven J. Raven J. C. Court J. H. (1998). *Manual for Raven’s Progressive Matrices and Vocabulary Scales. Section 2: The Coloured Progressive Matrices.* San Antonio, TX: Harcourt Assessment.

[B65] Raven J. C. Court J. H. Raven J. (1984). *Manual for Raven’s Progressive Matrices and Vocabulary Scales.* London: H.K. Lewis.

[B66] Reeve R. Reynolds F. Humberstone J. Butterworth B. (2012). Stability and change in markers of core numerical competencies. *J. Exp. Psychol. Gen.* 141 649–666. 10.1037/a0027520 22409662

[B67] Rittle-Johnson B. Siegler R. S. Alibali M. W. (2001). Developing conceptual understanding and procedural skill in mathematics: an iterative process. *J. Educ. Psychol.* 93 346–362. 10.1037/0022-0663.93.2.346

[B68] Rourke B. P. (1988). Socioemotional disturbances of learning disabled children. *J. Consult. Clin. Psychol.* 56:801. 10.1037/0022-006X.56.6.801 3060497

[B69] Ryoo J. H. Molfese V. J. Brown E. T. Karp K. S. Welch G. W. Bovaird J. A. (2015). Examining factor structures on the Test of Early Mathematics Ability - 3: A longitudinal approach. *Learn. Individ. Differ.* 41 21–29. 10.1016/j.lindif.2015.06.003

[B70] Sarnecka B. W. Carey S. (2008). How counting represents number: what children must learn and when they learn it. *Cognition* 108 662–674. 10.1016/j.cognition.2008.05.007 18572155

[B71] Sasanguie D. Göbel S. M. Moll K. Smets K. Reynvoet B. (2013). Approximate number sense, symbolic number processing, or number-space mappings: what underlies mathematics achievement? *J. Exp. Child Psychol.* 114 418–431. 10.1016/j.jecp.2012.10.012 23270796

[B72] Sasanguie D. Van den Bussche E. Reynvoet B. (2012). Predictors for mathematics achievement? evidence from a longitudinal study. *Mind Brain Educ.* 6 119–128. 10.1111/j.1751-228X.2012.01147.x

[B73] Schleifer P. Landerl K. (2011). Subitizing and counting in typical and atypical development. *Dev. Sci.* 14 280–291. 10.1111/j.1467-7687.2010.00976.x 22213901

[B74] Schmitt S. A. Geldhof G. J. Purpura D. J. Duncan R. McClelland M. M. (2017). Examining the relations between executive function, math, and literacy during the transition to kindergarten: a multi-analytic approach. *J. Educ. Psychol.* 109 1120–1140. 10.1037/edu0000193

[B75] Scüzs D. Devine A. Soltesz F. Nobes A. Gabriel F. (2014). Cognitive components of a mathematical processing network in 9-year-old children. *Dev. Sci.* 17 506–524. 10.1111/desc.12144 25089322 PMC4253132

[B76] Tolar T. D. Fuchs L. Fletcher J. M. Fuchs D. Hamlett C. L. (2016). Cognitive profiles of mathematical problem solving learning disability for different definitions of disability. *J. Learn. Disabil.* 49 240–256. 10.1177/0022219414538520 24939971 PMC4269584

[B77] Ullman J. B. (2001). “Structural equation modeling,” in *Using Multivariate Statistics*, 4th Edn, eds Tabachnick B. G. Fidell L. S. (Needham Heights, MA: Allyn & Bacon), 653–771.

[B78] Van de Weijer-Bergsma E. Kroesbergen E. H. Van Luit J. H. (2015). Verbal and visual-spatial working memory and mathematical ability in different domains throughout primary school. *Mem. Cogn.* 43 367–378. 10.3758/s13421-014-0480-4 25377509 PMC4555215

[B79] Vanbinst K. Ghesquiere P. De Smedt B. (2014). Arithmetic strategy development and its domain-specific and domain-general cognitive correlates: a longitudinal study in children with persistent mathematical learning difficulties. *Res. Dev. Disabil.* 35 3001–3013. 10.1016/j.ridd.2014.06.023 25124698

